# The Effect of Different Formulations of Praziquantel in Reducing Worms in the Prepatent Period of Schistosomiasis in Murine Models

**DOI:** 10.3389/fpubh.2022.848633

**Published:** 2022-05-27

**Authors:** Érica Tex Paulino, Monique Ribeiro de Lima, Alessandra Lifsitch Viçosa, Cleber Hooper da Silva, Claudio Javier Salomon, Daniel Andrés Real, Dario Leonardi, Clélia Christina Mello Silva, Antonio Henrique Almeida de Moraes Neto

**Affiliations:** ^1^Laboratory of Innovations in Therapies, Teaching and Bioproducts, Oswaldo Cruz Institute, Oswaldo Cruz Foundation (LITEB/IOC/FIOCRUZ), Rio de Janeiro, Brazil; ^2^Laboratory of Environmental Health Evaluation and Promotion, Oswaldo Cruz Institute, Oswaldo Cruz Foundation (LAPSA/IOC/FIOCRUZ), Rio de Janeiro, Brazil; ^3^Tropical Medicine Program, Oswaldo Cruz Institute, Oswaldo Cruz Foundation (IOC/FIOCRUZ), Rio de Janeiro, Brazil; ^4^Animal Experimentation Center, Oswaldo Cruz Institute, Oswaldo Cruz Foundation (IOC/FIOCRUZ), Rio de Janeiro, Brazil; ^5^Laboratory of Experimental Pharmacotechnics, Farmanguinhos, Oswaldo Cruz Foundation, Rio de Janeiro, Brazil; ^6^Institute of Science and Technology in Biomodels, Oswaldo Cruz Foundation, Rio de Janeiro, Brazil; ^7^Faculty of Biochemical and Pharmaceutical Sciences, University of Rosario, Rosario, Argentina; ^8^Institute of Chemistry of Rosario—National Research Council Scientific and Techniques (IQUIR-CONICET), Rosario, Argentina

**Keywords:** schistosomiasis, Praziquantel (PZQ), prepatent infection, *Schistosoma mansoni* (*S. mansoni*), nanoencapsulated

## Abstract

Schistosomiasis is a widely distributed parasitic disease and one of the most important neglected tropical diseases globally, for which Praziquantel® (PZQ) is the only available treatment. In this context, tests with new PZQ formulations become relevant for disease control. This study evaluated the effects of PZQ treatment in the prepatent phase of schistosomiasis using two formulations: nanoencapsulated (PZQ-NANO) and active pharmaceutical ingredient (PZQ-API). Five experimental groups were established, for which the following serological parameters were evaluated: ALT, AST, ALP, and TP. Animals treated with PZQ-API at 15 and 30 days post-infection showed decreased eggs per gram of feces (EPG) compared to untreated infected animals. The same animals showed reductions of 63.6 and 65.1%, respectively, at 60 days post-infection. Animals treated with PZQ-NANO experienced no significant changes in EPG at any time of observation. Animals treated with either PZQ-API or PZQ-NANO had higher ALT and AST levels in the patent period (60 and 90 days post-infection). Treatment with PZQ, either API or NANO, at 15 days post-infection reduced AST, ALT, and TP levels. It is concluded that prepatent treatment with PZQ-API can reduce the parasite load of infected animals and that treatment at 15 days post-infection can prevent increased serum levels of ALT, AST, and TP.

## Introduction

The World Health Organization (WHO) considers schistosomiasis to be one of the most important neglected tropical diseases. It affects more than 290 million people globally (1) and has chronic development that causes debilitating morbidity (2). This total, however, reaches ~440 million people when individuals cured of infection, but with remaining residual morbidity, are included (3). According to the Brazilian Ministry of Health (4), 1.5 million Brazilians are at risk of contracting the disease because they live in endemic areas. Between 2020 and 2021, the period corresponding to the COVID-19 pandemic, 212 hospitalizations and seven deaths from schistosomiasis were registered in the country, with cases being more frequent in the Northeast and Southeast regions (5).

The current recommendation for the control of schistosomiasis is based on the use of Praziquantel® (PZQ), not only because of its action in reducing disease prevalence and morbidity, but also for its schistosomicidal activity (6). However, the characteristic pathogenesis of the disease, namely hepatic periovular granuloma, still remains even with treatment since eggs of *S. mansoni* are carried to other organs *via* blood flow (7).

Although several studies have demonstrated the efficacy of PZQ against adult worms, larval forms are insensitive to its chemotherapeutic action (8–11) due to its marked evolutionary asynchronism (12–14). A treatment with proven effectiveness in the prepatent period of schistosomal infection would significantly benefit human health, since without the development of adult forms there would be no egg production and, consequently, no formation of granulomas nor the development of other comorbidities (14).

Nanotechnology has recently been used as an effective strategy for drug delivery to predetermined targets. The method presents a series of advantages such as increased bioavailability, decreased systemic drug absorption, reduced side effects, accurate drug targeting, and increased time of action in the body, as the release of the bioactive agent is gradual and controlled (15–17).

The search for efficacy in treating mass infections, or for new formulations containing PZQ or even new drugs, becomes relevant to achieving adequate control of schistosomiasis in endemic areas. Thus, this study aimed to evaluate the effects of PZQ treatment in the prepatent phase of *S. mansoni* infection under experimental conditions using two different formulations: nanoencapsulated and active pharmaceutical ingredient only.

## Methods

Two-hundred and eighty female mice, *Mus musculus* (*Swiss Webster* strain), ~4 weeks old (20–25 g), were kept, infected, and euthanized in a certified vivarium following IOC/FIOCRUZ bioethics and biosafety rules. All animals were maintained with a 12-h light/12-h dark cycle, humidity control, environmental enrichment and feed (previously autoclaved) and water provided *ad libitum*. Individual mice were experimentally infected subcutaneously with 0.3 ml of a suspension of 150 (± 10) cercariae (within 2 h after their release from snails) in dechlorinated water (18). The LE strain of *S. mansoni* used in this experiment came from the Schistosomiasis Reference Service of the Malacology Laboratory of IOC/FIOCRUZ. All animals were infected only once and followed until blood collection for serological exams.

The formulations of PZQ used were “pharmaceutical active ingredient” (PZQ-API) and nanoencapsulated (PZQ-NANO). PZQ-NANO was produced by nanoprecipitation, using a polymer (Eudragit E100) contained in an organic phase and PZQ in an aqueous phase containing a stabilizer to guarantee the use of this nanoparticle in various drug formulations (19, 20). Polyvinyl alcohol (PVA) was used as an anti-aggregating agent to prevent coalescence of nanoparticles. The obtained nanosuspension was then dried by “Spray Drying” using spray-drying equipment (Buchi B- 290), with Maltodextrin (dextrose equivalent 4.0–7.0) as a diluent, to obtain a stable formulation that guarantees stability during experiments. The qualitative and quantitative composition of the nanoparticles was 20.8% PZQ (0.208 mg PZQ in 1 mg of nanoparticulate material), 20.8% Eudragit E, 8.4% PVA, and 50% Maltodextrin. Both formulations used in this study were administered to mice in aqueous suspension *via* a single dose of 400 mg/Kg by gavage. The chosen dosage was based on studies attesting to its effectiveness in treating schistosomal infection in mice (21, 22).

**Figure 1:**
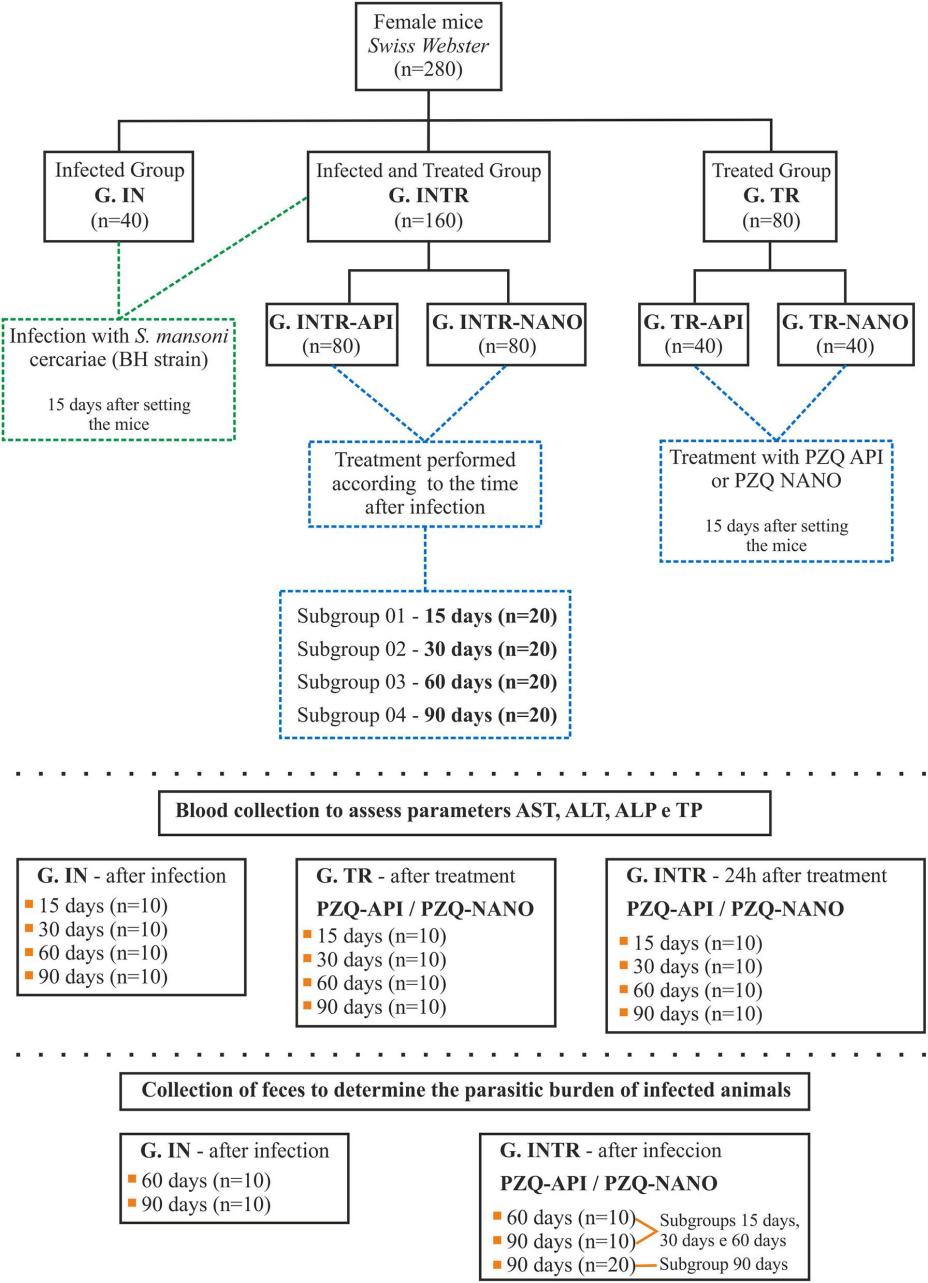
Flowchart of the experimental design of the study.

The mice were randomly allocated to five experimental groups (Figure 1) and acclimatized for 15 days. Animals of the infected and untreated group (IN) and of the infected and treated group (INTR) were then infected, and those of uninfected and treated group (TR) and INTR were treated with PZQ-API or PZQ-NANO. Thus the five experimental groups were: G. IN, G. TR-API, G. TR-NANO, G. INTR-API and G. INTR-NANO. Observations were made at 15, 30, 60, and 90 days post-infection (p.i.) to analyze the behavior of the infection and treatment in the prepatent (15 and 30 days) and patent (60 and 90 days) stages of infection. Mice belonging to groups INTR-API and INTR-NANO were organized into four subgroups with 20 animals each, which were treated with PZQ (API or NANO, respectively) at 15, 30, 60, and 90 days p.i.

All serological tests were performed at the Technological Platform—Laboratory Animals/Clinical Analyses based at Institute of Science and Technology in Biomodels (ICTB/FIOCRUZ). The following biochemical parameters were analyzed: Alkaline Phosphatase (ALP; reference value: 62 to 209 U/L), Alanine Aminotransferase (ALT; reference value: 28 to 132 U/L), Aspartate Aminotransferase (AST; reference value: 59 to 247 U/L) and Total Proteins (TP; reference value: 3.6 to 6.6 g/dL). Blood collections were performed by cardiac puncture, using a disposable syringe with a 20 x 0.55 mm needle, of animals previously anesthetized with 100 mg/kg of ketamine associated with 10 mg/kg of xylazine hydrochloride *via* intraperitoneal, with anesthesia depth being verified by sensitivity test using painful stimuli. Collected blood was transferred to a 500-μl microtube with gel and clot activator and centrifuged at 13,000 RPM, for 3 mins, at room temperature. The serum was then transferred to a 0.5 ml Eppendorf and sent to the clinical analysis platform of ICTB/FIOCRUZ for serological analysis. Ten infected animals (G. IN) and ten animals treated with PZQ (G. TR-NANO and G. TR-API) were selected at each observation time (15, 30, 60, and 90 days p.i. or treatment) for blood collection. In the infected and treated groups (INTR-NANO and INTR-API), blood was collected from 10 animals, 24 h post treatment with PZQ, at the same observation times. Animals that did not die after this procedure were euthanized by barbiturate overdose: intracardiac administration of 2.5% sodium thiopental (300 mg/kg) by the responsible veterinarian, according to the protocol approved by the Animal Ethics Commissions (CEUA).

Parasitic load was determined as the number eggs per gram of feces (EPG) using the Kato-Katz method (23). Feces were collected from infected mice at 60 and 90 days p.i. (prior to treatment for those that received treatment at 60 and 90 days p.i.). Feces were collected in the morning, individually and for an hour to avoid animal stress, after which mice were returned to their respective cages. Three slides were prepared for each feces sample. Counts were made under a light microscope and the arithmetic mean of eggs per feces sample was calculated and multiplied by 24 (factor) to obtain EPG. Parasitic load was classified according to the criteria adopted by the WHO (24), and referenced in the protocol of the Kato-Katz Kit, as follows: 1–99 EPG = light intensity; 100–399 EPG = moderate intensity; ≥400 EPG = heavy intensity.

The biochemical parameters of serological tests and the results of the EPG were compared between the different groups of both pharmaceutical formulations containing PZQ for the evaluation of the therapeutic responses of the mentioned formulations. The Mann-Whitney-Wilcoxon test was used to assess differences between groups treated with PZQ-NANO and PZQ-API, using the group of untreated infected animals (G. IN) and the group of treated and uninfected animals (G. TR-NANO and G. TR-API) as controls. All results were input into Microsoft Access database software and analyzed using R version 4.0.2 (25) with a significance level (α) of 5%.

## Results

In all, 120 feces samples were collected from animals at 60 days p.i. and 110 from animals at 90 days p.i.

Table 1 presents the results of the Mann-Whitney-Wilcoxon paired test for EPG at 60 and 90 days p.i. The IN control group and subgroups INTR-API 15 days, INTR-API 30 days and INTR-API 60 days showed significant decreases in EPG at 90 days p.i. The subgroups INTR-API 15 days and INTR-API 30 days, both treated in the prepatent period of infection, showed EPG reductions of 63.6% [1–(78.8/216.9)] and 65.1% [1–(75.6/216.9)], respectively, at 60 days p.i., when compared to the IN control group. This reduction was also observed at 90 days p.i., with the subgroups INTR-API 15 days and INTR-API 30 days showing EPG reductions of 72.3% [1–(25.6/92.4)] and 70.1% [1–(27.6/92.4)], respectively, compared to the IN control group. There was no significant difference in EPG for PZQ-NANO treated groups at both 60 and 90 days p.i., compared to the IN control group. However, animals treated with PZQ-API having a greater reduction in EPG at 90 days p.i. than animals treated with PZQ-NANO (Supplementary Figure 1).

**Table 1:** Average amount of eggs per gram of feces (EPG) obtained from mice infected with *S. mansoni* in two periods: 60 days and 90 days after schistosomal infection.

**GROUPS**	**60 days p.i**.			**90 days p.i**.			**EPG reduction (%)**	***P*-value**
	***N***	**EPG (mean)**	**Intensity of infection**	***N***	**EPG (mean)**	**Intensity of infection**		
IN	60	216.9^a^	Moderate	50	92.4^a^	Light	57.4	0.000*
INTR-API 15 DAYS	10	78.8	Light	10	25.6	Light	67.5	0.005*
INTR-API 30 DAYS	10	75.6	Light	10	27.6	Light	63.5	0.022*
INTR-API 60 DAYS	10	130^a^	Moderate	10	42.8	Light	67.0	0.014*
INTR-NANO 15 DAYS	10	180.8	Moderate	10	210.4	Moderate	−16.3^b^	0.722
INTR-NANO 30 DAYS	10	218	Moderate	10	311	Moderate	−42.6^b^	0.250
INTR-NANO 60 DAYS	10	90.4^a^	Light	10	224.4	Moderate	−148.2^b^	0.138
Total	120			110				

a EPG result for untreated infected animals. The P-value for the paired Mann-Whitney-Wilcoxon test. *Significant values for P < 0.05.

b *Negative values demonstrate an increase in EPG*.

**Figure 2:**
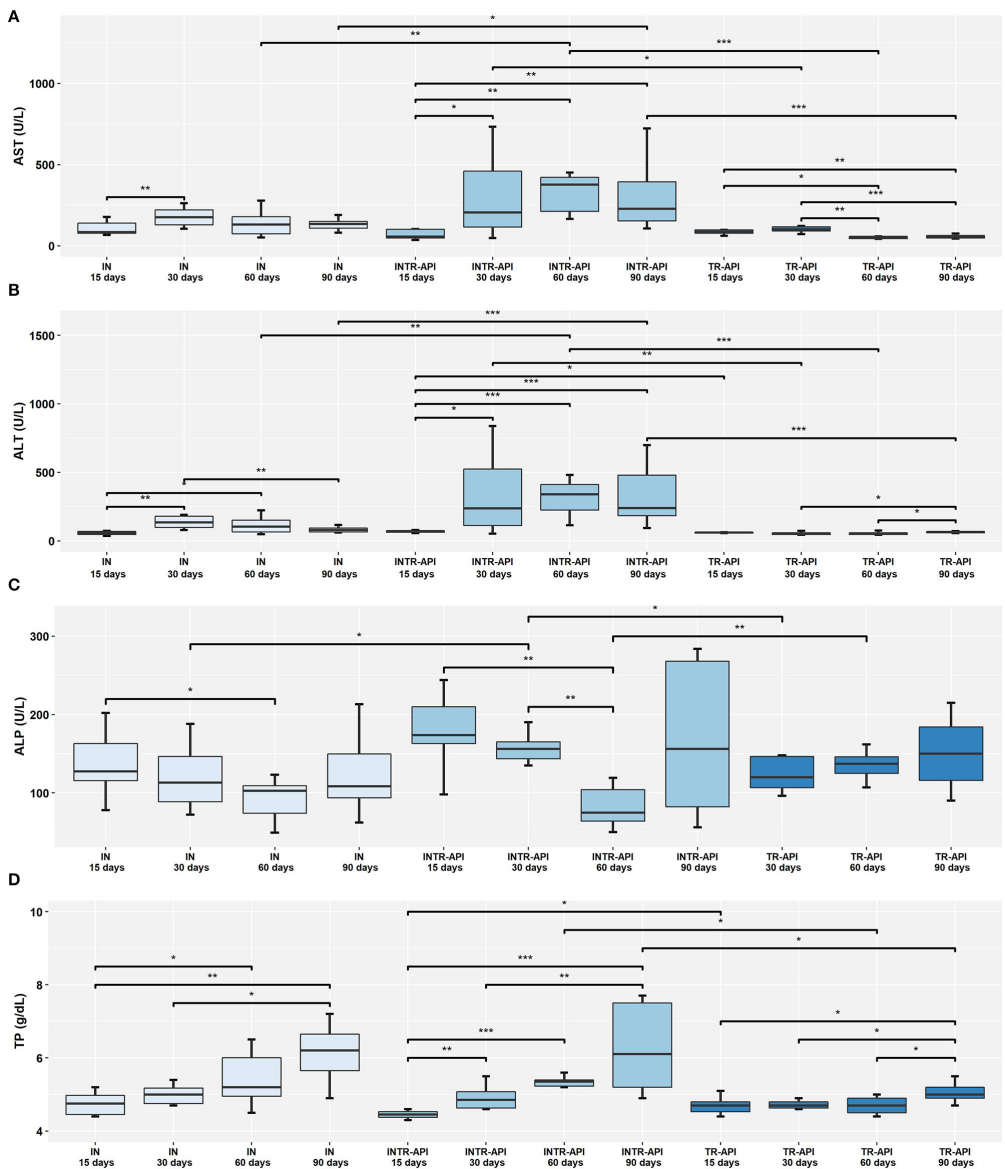
Comparison of the results of serological parameters between the IN, INTR-API and TR-API groups. **(A)** AST, Aspartate Transaminase; **(B)** ALT, Alanine Transaminase; **(C)** ALP, Alkaline Phosphatase; and **(D)** TP, Total Proteins. Mann-Whitney-Wilcoxon test: * *P* < 0.05, **P < 0.01, ***P < 0.001.

**Figure 3:**
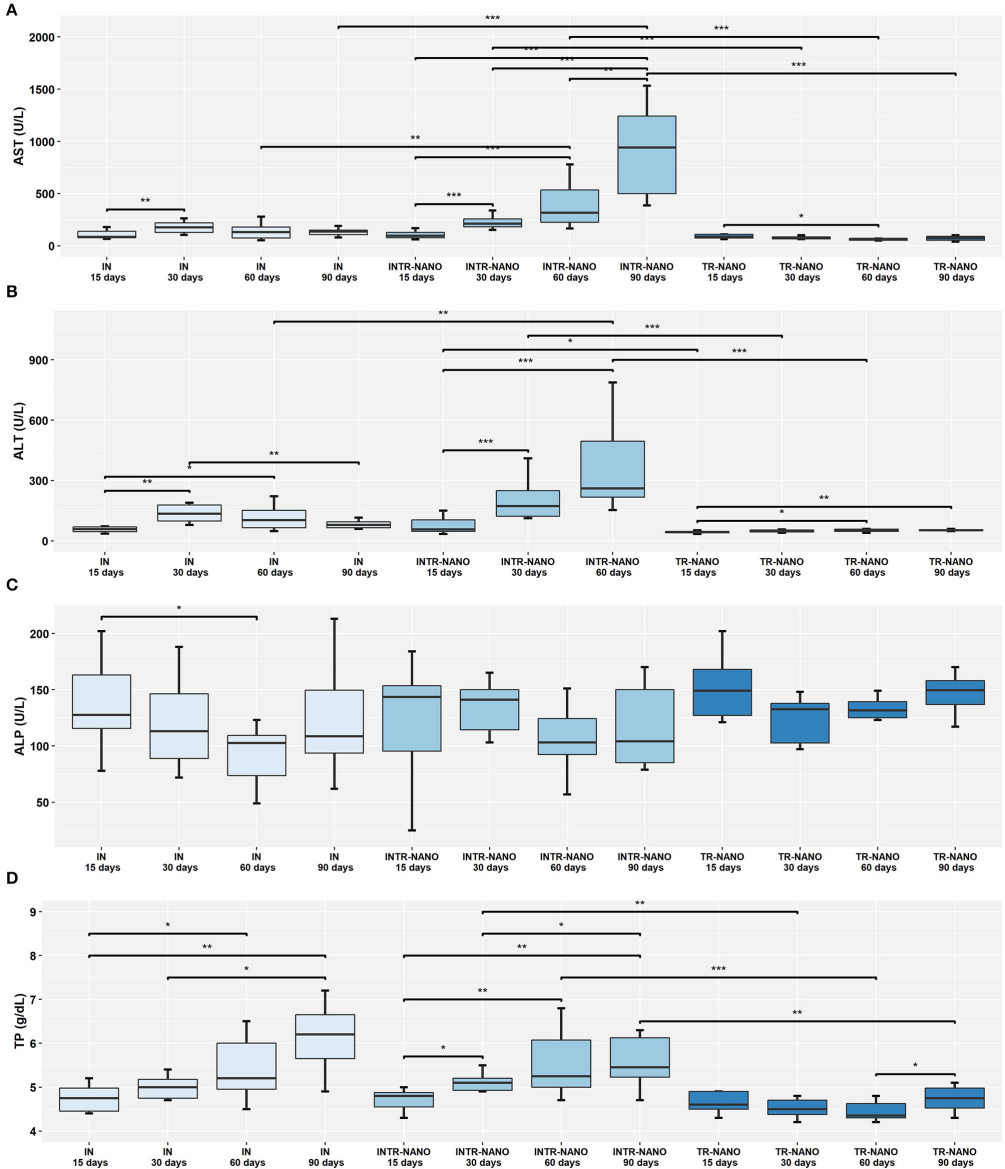
Comparison of the results of serological parameters between the IN, INTR-NANO, and TR-NANO groups. **(A)** AST, Aspartate Transaminase; **(B)** ALT, Alanine Transaminase; **(C)** ALP, Alkaline Phosphatase; and **(D)** TP, Total Proteins. Mann-Whitney-Wilcoxon test: *P < 0.05, **P < 0.01, ***P < 0.001.

Analysis of animals infected and treated with either PZQ-API or PZQ-NANO revealed significantly lower serum levels of AST, ALT and TP for subgroups INTR-API 15 days (Figures 2A,B,D) and INTR-NANO 15 days (Figures 3A,B,D). For infected and untreated animals, IN 15 days had significantly lower serum AST levels than IN 30 days (*P* < 0.01) (Figure 2A) and lower serum ALT levels than IN 30 days (*P* < 0.01) and IN 60 days (*P* < 0.05) (Figure 2B).

Comparison of groups infected and treated with either PZQ-API or PZQ-NANO for parameters ALT, AST, ALP and TP (Supplementary Figure 2) revealed that ALT and TP did not differ significantly between subgroups with the same treatment time but of different drug treatments (PZQ-API or PZQ-NANO) (Supplementary Figures 2B,D). There were lower serum AST levels for subgroup INTR-API 90 days compared to subgroup INTR-NANO 90 days (*P* < 0.01) (Supplementary Figure 2A), while serum ALP levels were significantly higher for subgroup INTR-API 15 days compared to subgroup INTR-NANO 15 days (*P* < 0.05) (Supplementary Figure 2C).

Analysis of serum levels of ALT and AST between the IN control group and groups INTR-API and INTR-NANO revealed a significant increase (*P* < 0.05) for animals treated during the patent period of infection, that is, 60 and 90 days p.i. (Figures 2A,B, 3A,B). Animals of the groups TR-API and TR-NANO maintained significantly lower serum levels of ALT and AST compared to groups INTR-API and INTR-NANO, respectively, showing that only treatment with the PZQ formulations evaluated in this study in uninfected animals did not is related to increased serum levels of these enzymes (Figures 2A,B, 3A,B).

There were higher serum levels of ALP at 15 days p.i. and lower levels at 60 days p.i. for both IN (*P* < 0.05) and INTR-API (*P* < 0.01) (Figure 2C). The results remained within the reference range (62 to 209 U/L), except for two animals in the subgroup “INTR-API 15 days” (244 U/L and 219 U/L). Serum levels of this enzyme was increased in both IN and INTR-API at 90 days p.i., with three animals in INTR-API having values above the reference range (i.e., 690 U/L, 284 U/ L and 268 U/L). Animals of INTR-NANO and TR-NANO did not differ in serum levels of ALP at any of the observed times/subgroups, nor when compared to the IN control group (Figure 3C).

ALT results for the subgroup INTR-NANO 90 days and the group TR-API 15 days were not included in the analyses because they were not available due to a technical equipment error at the time of analysis.

## Discussion

The elimination of *Schistosomiasis mansoni* as a public health problem has been the focus of disease control in the 21st century. As a solution, WHO (24) has proposed several actions to control transmission, including treatment with PZQ in endemic areas. This plan recommends treatment of schistosomiasis without prior diagnosis for active populations in endemic areas with transmission above 25% (26, 27). This strategy is known to provide treatment to people who are not only in the patent period of infection, but also in the prepatent period and without infection, which may or may not lead to drug resistance.

The present study verified that treatment with PZQ-API, which is similar to the formulation used for the treatment in endemic areas of schistosomiasis in Brazil, is efficient in the treatment of schistosomal infection in the pre-patent period of infection, with significant reduction in EPG. These results validate the epidemiological strategy of the Brazilian Ministry of Health, and in line with the WHO, and refutes some previous work indicating that PZQ acts only during the patent period (28–33). These results become essential since early treatment could reduce morbidities associated with schistosomiasis, such as chronic anemia, liver fibrosis, portal hypertension, ascites, hepatic encephalopathy, and cognitive deficit (very common in children), among others (26, 34, 35). They may also lead to new strategies, such as treatment of school-age children, the primary victims of schistosomiasis (36–38) because they are more exposed to parasite transmission due to their leisure activities.

The present study also evaluated the PZQ-NANO therapy to offer an alternative for the treatment of *Schistosomiasis mansoni*. Nanoencapsulated pharmaceutical formulations represent an increasingly used drug delivery system, mainly for the treatment of some neglected diseases (39–45). Studies have pointed out that nanoformulations improve drug stability and bioavailability and direct it to the target zone, as well as optimize therapy and reduce adverse effects (41, 42). Both *in vivo* and *in vitro* studies (42–45) have demonstrated the effectiveness of nanoformulations containing PZQ at reducing the parasite burden of adult worms and improving liver damage in mice, while also damaging the integument of *S. mansoni* adults. Furthermore, encapsulation of PZQ reduces cytotoxicity, which indicates that this drug distribution system holds promise for the control of schistosomiasis (42–45). However, contrary to what can be observed in the literature, the nanoencapsulated formulation developed in Argentina and sent to Brazil for tests was not effective in treating schistosomal infection. This finding may be associated with the rigidity of the capsule of this formulation, which may have caused prolonged drug release in the gastrointestinal system, resulting in decreased concentrations absorbed by the intestines and, consequently, the action of the drug on adult worms.

The liver plays an essential role in schistosomal infection because it is where schistosomal cells mature to their adult form (46). Also, the induced formation of periovular granulomas in the liver by the immune response of the definitive host in response to the presence of worm eggs trapped in the walls of hepatic vessels and the development of periportal hepatic fibrosis characterize severe lesions of the organ that generate changes in liver enzymes (47, 48). For this reason, it is extremely important to evaluate liver function when determining the efficiency of PZQ at treating schistosomal infection. The liver enzymes evaluated in the present study were used to indicate possible lesions in hepatocytes and bile ducts of the liver.

According to the present results, the group of infected and untreated animals and the groups of infected animals treated with PZQ-API experienced higher levels of the enzyme ALP at 15 days p.i., after which it decreased until reaching its lowest levels at 60 days p.i. At 90 days p.i., serum ALP levels increased again, extensively, to surpass the reference range. ALP is routinely evaluated by hepatogram and is a parameter of great importance in diagnosing liver diseases. Some studies have related decreased serum ALP to an anemia characteristic of infection by *Schistosomiasis mansoni* (49, 50). Alternatively, increased ALP may be associated with changes in the intrahepatic bile duct, resulting from periportal fibrosis and/or periovular granuloma (51, 52). The results obtained here match the phases of *S. mansoni* in the definitive host. During the prepatent period, schistosomal cells are in the bloodstream feeding on red blood cells, while in the patent period, the adult worms are lodged in the hepatic and intestinal systems causing pathological changes in those organs.

The serum levels of TP were similar among the groups of infected animals (G. IN, G. INTR-API and G. INTR-NANO), showing lower results at 15 days p.i. and reaching the highest values at 90 days p.i. The increase in TP serum levels may be associated with a decompensated increase in globulins due to an aggravation of infection, which is observed mainly in animals with high parasite load (53).

The parameters ALT and AST were the most sensitive for pointing out differences in their plasma concentrations between the groups infected and treated with PZQ (INTR-API and INTR-NANO) and the most used indicators of hepatic lesions in the literature (50, 54). The animals treated with PZQ-API and PZQ-NANO at 15 days p.i. presented lower serum levels of ALT and AST than those infected and treated at the other times (30, 60 and 90 days p.i.), but similar to infected and untreated animals at 15 days p.i. (G. IN 15 days). For these same parameters, treatment of uninfected animals with PZQ-API or PZQ-NANO conferred lower serum levels than for infected animals. However, treatment at 30 days, 60 days and 90 days p.i. (PZQ-NANO and PZQ-API) caused a significant increase in ALT and AST when compared to the control group of infected and untreated animals (G. IN) and uninfected and treated animals (G. TR-API and G. TR-NANO). The increase in serum levels of these enzymes is related to necrosis of liver tissue caused by the formation of peri-ovular fibrosis and granulomas (55, 56). No references could be found in the literature that may explain the increase in serum levels of these enzymes after treatment. However, some synergistic effect may have occurred in liver metabolism due to *S. mansoni* infection and treatment with PZQ. It was also shown that treatment with PZQ-API in the prepatent period had less liver damage than treatment in the patent period. This can be explained by the biological cycle of the parasite. In the prepatent period (15 days), the adult worms of *S. mansoni* are not yet found in the liver and therefore have not mated or laid eggs. It should be noted that pathogenic liver lesions of schistosomiasis are associated with the presence of parasite eggs in the liver.

## Conclusions

This study demonstrated that treatment with PZQ-API at 15 days and 30 days after schistosomal infection (pre-patent period) significantly reduces the number of eggs per gram of feces in infected mice. Serum levels of ALT, AST, and TP were lower in treated animals (with PZQ-API or PZQ-NANO) at 15 days p.i. than in animals treated at 30 days, 60 days, and 90 days p.i. Treatment with PZQ-NANO did not reduce EPG in infected animals in any period of the experiment. Therefore, more studies are needed to better understand the action mechanisms of PZQ on young forms of *S. mansoni* and develop more effective formulations with less side effects.

## Data Availability Statement

The raw data supporting the conclusions of this article will be made available by the authors, without undue reservation.

## Ethics Statement

The animal study was reviewed and approved by Animal Ethics Commissions of Instituto Oswaldo Cruz.

## Author Contributions

CM and ÉP developed the study design. ÉP, CM, and MR developed all the experiments with the mice. CSi did all the biochemical analyzes of the animals. CM and AM coordinated the project. AV and CSa developed the drugs and assisted with scientific support in this study. DR and DL were responsible for the formulation and characterization of the nanoencapsulated PZQ. All authors have reviewed the manuscript and contributed to the article and approved the submitted version.

## Funding

This work was carried out with the support of the Coordination for the Improvement of Higher Education Personnel—Brazil (CAPES)—Financing Code 001 and with the support of the POM/PAEF of the Laboratory of Innovations in Therapies, Teaching, and Bioproducts (IOC/Fiocruz) and the Laboratory for Assessment and Promotion of Environmental Health (IOC/Fiocruz).

## Conflict of Interest

The authors declare that the research was conducted in the absence of any commercial or financial relationships that could be construed as a potential conflict of interest.

## Publisher's Note

All claims expressed in this article are solely those of the authors and do not necessarily represent those of their affiliated organizations, or those of the publisher, the editors and the reviewers. Any product that may be evaluated in this article, or claim that may be made by its manufacturer, is not guaranteed or endorsed by the publisher.

## References

[1] WHO. Schistosomiasis. (2020). Available online at: https://www.who.int/news-room/fact-sheets/detail/schistosomiasis (accessed November 2, 2021).

[2] Bergquist NR Leonardo LR Mitchell GF. Vaccine-linked chemotherapy: can schistosomiasis control benefit from an integrated approach? Trends Parasitol. (2005) 21:112–7. 10.1016/j.pt.2005.01.001 15734657

[3] Colley DG Bustinduy AL Secor WE King CH. Human schistosomiasis. Lancet. (2014) 383:2253–64. 10.1016/S0140-6736(13)61949-2 24698483 PMC4672382

[4] Noya O Katz N Pointier JP Theron A Noya BA. (2015). “Schistosomiasis in America”, in Neglected Tropical Diseases—Latin America and the Caribbean, eds. C. Franco-Paredes and J.I. Santos-Preciado (Vienna: Springer), pp. 12–38.

[5] Ministério da Saúde (BR). Portal da Saúde SUS—Datasus. Informações da saúde—demográficas e socioeconômicas [Internet]. (2021). Disponível em: http://tabnet.datasus.gov.br/cgi/deftohtm.exe?sih/cnv/nruf.def [Acessado em 31 de dezembro de 2021].

[6] Olliaro P Seiler J Kuesel A Horton J Clark JN Don R. Potential drug development candidates for human soil-transmitted helminthiases. PLoS Negl Trop Dis. (2011) 5:e1138. 10.1371/journal.pntd.0001138 21695247 PMC3111745

[7] Katz N. “Terapêutica Clínica na Esquistossomose Mansoni” in: Schistosoma mansoni e esquistossomose: uma visão multidisciplinar, ed O.S. Carvalho (Rio de Janeiro, RJ: Editora FIOCRUZ), p. 849–70 (2008).

[8] Xiao SH Catto BA. Comparative in vitro and in vivo activity of racemic praziquantel and its levorotated isomer on *Schistosoma mansoni*. J Infect Dis. (1989) 159:589–92. 10.1093/infdis/159.3.589 2915173

[9] Doenhoff MJ Sabah AA Fletcher C Webbe G Bain J. Evidence for an immune-dependent action of praziquantel on *Schistosoma mansoni* in mice. Trans R Soc Trop Med Hyg. (1987) 81:947–51. 10.1016/0035-9203(87)90360-9 3140436

[10] Cioli D Pica-Mattoccia L. Praziquantel. Parasitol Res. (2003) 90:S3–9. 10.1007/s00436-002-0751-z 12811543

[11] Botros S Pica-Mattoccia L William S El-Lakkani N Cioli D. Effect of praziquantel on the immature stages of *Schistosoma haematobium*. Int J Parasitol. (2005) 32:1453–7. 10.1016/j.ijpara.2005.05.002 16002073

[12] Barbosa MA Pellegrino J Coelho PMZ Sampaio IBM. Quantitative aspects of the migration and evolutive asynchronism of *Schistosoma mansoni* in mice. Rev Inst Med Trop São Paulo. (1978) 20:121–32. 684324

[13] Faust EC Jones CA Hoffmam WA. Studies on schistosomiasis mansoni in Puerto Rico. III- Biological studies 2 The mammalian phase of the life cycle. P R Health Sci J. (1934) 10:133–96.

[14] Vimeiro AC Araújo N Katz NKusel JR Coelho PM. Schistogram changes after administration of antischistosomal drugs in mice at the early phase of *Schistosoma mansoni* infection. Mem Inst Oswaldo Cruz. (2013) 108:881–6. 10.1590/0074-0276130135 24271044 PMC3970651

[15] Suganya V Anuradha V. Microencapsulation and nanoencapsulation: a review. Int J Phar Clin Res. (2017) 9:233–9. 10.25258/ijpcr.v9i3.8324

[16] Eissa MM El-Azzouni MZ El-Khordagui LK Abdel Bary A El-Moslemany RM Abdel Salam SA. Evaluation of prophylactic efficacy and safety of praziquantel-miltefosine nanocombination in experimental Schistosomiasis mansoni. Acta Trop. (2020) 212:105714. 10.1016/j.actatropica.2020.105714 32950482

[17] Eissa MM El-Azzouni MZ El-Khordagui LK Abdel Bary A El-Moslemany RM Abdel Salam SA. Single oral fixed-dose praziquantel-miltefosine nanocombination for effective control of experimental schistosomiasis mansoni. Parasit Vectors. (2020) 13:474. 10.1186/s13071-020-04346-1 32933556 PMC7493353

[18] Peters PA Warren KS. A rapid method of infecting mice and other laboratory animals with Schistosoma mansoni: subcutaneous injection. J Parasitol. (1969) 55:558. 10.2307/3277297

[19] Molpeceres J Guzman M Aberturas MR Chacon M Berges L. Application of central composite designs to the preparation of polycaprolactone nanoparticles by solvent displacement. J Pharm Sci. (1996) 85:206–13. 10.1021/js950164r 8683450

[20] Quintanar-Guerrero D. Alle'mann E, Fessi H, Doelker E. Pseudolatex preparation using a novel emulsion-diffusion process involving direct displacement of partially water-miscible solvents by distillation. Int J of Pharm. (1999) 188:155–64. 10.1016/S0378-5173(99)00216-1 10518671

[21] Couto FFB Coelho PMZ Araújo N Kusel JR Katz N Jannotti-Passos LK. *Schistosoma mansoni*: a method for inducing resistance to praziquantel using infect *Biomphalaria glabrata* snails. Mem Inst Oswaldo Cruz. (2011) 106:153–7. 10.1590/S0074-02762011000200006 21537673

[22] Araújo N Souza CP Passos LKJ Simpson AJG Neto ED Pereira TR. Suscetibilidade aos agentes quimioterápicos de isolados de *Schistosoma mansoni* oriundos de pacientes tratados com oxamniquine e praziquantel e não curados. Rev Soc Bras Med Trop. (1996) 29:467–76. 10.1590/S0037-86821996000500010 8966311

[23] Katz N Chaves A Pellegrino J. A simple device for quantitative stool thick-smear technique in Schistosomiasis mansoni. Rev Inst Med Trop S Paulo. (1972) 14:397–400. 4675644

[24] World Health Organization. Prevention and control of schistosomiasis and soil-transmitted helminthiasis: report of a WHO expert committee. Technical Report Series. Geneva. Report No.: 912. (2002). 12592987

[25] R Core Team. R: A Language and Environment for Statistical Computing Version 3.5.2. Vienna, Austria: R Foundation for Statistical Computing (2018).

[26] Ministério da Saúde (BR). Vigilância da esquistossomose mansônica: diretrizes técnicas. Brasília: Ministério da Saúde; 4ª edição (2014).

[27] World Health Organization. Schistosomiasis: progress report 2001-−2011, strategic plan 2012-−2020. Geneva: World Health Organization, pp. 1–80 (2013).

[28] Gönnert R Andrews P. A new broad-spectrum antischistosomal agent. Z Parasitenkd. (1977) 52:129–50. 10.1007/BF00389899 410178

[29] Sabah AA Fletcher C Webbe G Doenhoff MJ. *Schistosoma mansoni*: chemotherapy of infections of different ages. Exp Parasitol. (1986) 61:294–303. 10.1016/0014-4894(86)90184-0 3086114

[30] Pica-Mattoccia L Cioli D. Sex-and stage-related sensitivity of *Schistosoma mansoni* to in vivo and in vitro praziquantel treatment. Int J Parasitol. (2004) 34:527–33. 10.1016/j.ijpara.2003.12.003 15013742

[31] Becker B Mehlhorn H Andrews P Thomas H Eckert J. Light and electron microscopic studies on the effect of praziquantel on *Schistosoma mansoni, Dicrocoelium dendriticum*, and *Fasciola hepatica* (Trematoda) in vitro. Z Parasitenkd. (1980) 63:113–28. 10.1007/BF00927527 7456640

[32] Mehlhorn H Becker B Andrews P Thomas H Frenkel JK. In vivo and in vitro experiments on the effects of praziquantel on *Schistosoma mansoni*. a light and electron microscopic study. Arzneimittelforschung. (1981) 31:544–54. 7195245

[33] Cioli D Pica-Mattoccia L Archer S. Antischistosomal drugs: past, present and future? Pharmacol Ther. (1995) 68:35–85. 10.1016/0163-7258(95)00026-7 8604437

[34] Bustinduy AL Friedman JF Kjetland EF Ezeamama AE Kabatereine NB Stothard JR. Expanding praziquantel (PZQ) access beyond mass drug administration programs: paving a way forward for a pediatric PZQ formulation for schistosomiasis. PLoS Negl Trop Dis. (2016) 10:e0004946. 10.1371/journal.pntd.0004946 27658198 PMC5033572

[35] Weerakoon KGAD Gobert GN Cai P McManus DP. Advances in the diagnosis of human schistosomiasis. Clinic Microbiol Rev. (2015) 28:939–67. 10.1128/CMR.00137-14 26224883 PMC4548261

[36] Vennervald B Ouma J Butterworth A. Morbidity in schistosomiasis: assessment, mechanisms and control. Parasitol Today. (1998) 14:385–90. 10.1016/S0169-4758(98)01311-8 17040825

[37] Kabatereine N Kemijumbi J Ouma J Kariuki H Richter J Kadzo H. Epidemiology and morbidity of *Schistosoma mansoni* infection in a fishing community along Lake Albert in Uganda. Trans R Soc Trop Med Hyg. (2004) 98:711–8. 10.1016/j.trstmh.2004.06.003 15485701

[38] Salam RA Maredia H Das JK Lassi ZS Bhutta ZA. Community-based interventions for the prevention and control of helmintic neglected tropical diseases. Infect Dis Poverty. (2014) 3:23. 10.1186/2049-9957-3-23 25114793 PMC4128617

[39] Silva LD Lima NF Arrua EC Salomon CJ Vinaud MC. In vivo treatment of experimental neurocysticercosis with praziquantel nanosuspensions—a metabolic approach. Drug Deliv Transl Res. (2018) 18:7. 10.1007/s13346-018-0576-7 30117119

[40] Islan GA Durán M Cacicedoa ML Nakazatod G Kobayashi RKT Martinez DST. Nanopharmaceuticals as a solution to neglected diseases: is it possible? Acta Trop. (2017) 170:16–42. 10.1016/j.actatropica.2017.02.019 28232069

[41] Ghosh S Ghosh S Sil PC. Role of nanostructures in improvising oral medicine. Toxicol Rep. (2019) 6:358–68. 10.1016/j.toxrep.2019.04.004 31080743 PMC6502743

[42] Amara RO Ramadan AA El-Moslemany RM Eissa MM El-Azzouni MZ El-Khordagui LK. Praziquantel-lipid nanocapsules: an oral nanotherapeutic with potential Schistosoma mansoni tegumental targeting. Int J Nanomedicine. (2018) 13:4493–505. 10.2147/IJN.S167285 30122922 PMC6084080

[43] Kolenyak-Santos F Garnero C de Oliveira RN de Souza AL Chorilli M Allegretti SM. Nanostructured lipid carriers as a strategy to improve the in vitro schistosomiasis activity of praziquantel. J Nanosci Nanotechnol. (2015) 15:761–72. 10.1166/jnn.2015.9186 26328440

[44] Mishra A Swain RK Mishra SK Panda N Sethy K. Growth performance and serum biochemical parameters as affected by nano zinc supplementation in layer chicks. Indian J Anim Nutr. (2014) 31:384–8.

[45] Yang L Geng Y Li H Zhang Y You J Chang Y. Enhancement the oral bioavailability of praziquantel by incorporation into solid lipid nanoparticles. Pharmazie. (2009) 64:86–9. 10.1691/ph.2009.8140 19320279

[46] Wilson RA. The saga of schistosome migration and attrition. Parasitology. (2009) 136:1581–92. 10.1017/S0031182009005708 19265564

[47] Warren KS. The Secret of the Immunopathogenesis of Schistosomiasis: IN VIVO Models. Immunol Rev. (1982) 61:189–213. 10.1111/j.1600-065X.1982.tb00377.x 7037609

[48] Wynn TA Thompson RW Cheever AW Mentink-Kane MM. Immunopathogenesis of schistosomiasis. Immunol Rev. (2004) 201:156–67. 10.1111/j.0105-2896.2004.00176.x 15361239

[49] Thapa BR Walia A. Liver function tests and their interpretation. Indian J Pediatr. (2007) 74:663–71. 10.1007/s12098-007-0118-7 17699976

[50] Jatsa HB Kenfack CM Simo DN Feussom NG Nkondo ET Tchuem Tchuente LA. Schistosomicidal, hepatoprotective and antioxidant activities of the methanolic fraction from Clerodendrum umbellatum Poir leaves aqueous extract in *Schistosoma mansoni* infection in mice. BMC Complement Altern Med. (2015) 15:248. 10.1186/s12906-015-0788-z 26205948 PMC4513613

[51] Leite LAC Domingues ALC Lopes EP Ferreira RCS Filho AAP Fonseca CSM. Relationship between splenomegaly and hematologic findings in patients with hepatosplenic schistosomiasis. Rev Bras Hematol Hemoter. (2013) 35:332–6. 10.5581/1516-8484.20130098 24255616 PMC3832313

[52] Leite LA Pimenta Filho AA Ferreira RD Fonseca CS Santos BS Montenegro SM. Splenectomy improves hemostatic and liver functions in hepatosplenic schistosomaisis mansoni. PLoS One. (2015) 10:e0135370. 10.1371/journal.pone.0135370 26267788 PMC4534302

[53] Atta AM. Esquistossomose mansônica II — Evolução dos níveis de proteínas séricas e do perfil eletroforético por técnicas de imunoeletroforese quantitativa. Rev Saude Publ. (1981) 15:194–204. 10.1590/S0034-89101981000200004 7323662

[54] Elhenawy AA Ashour RH Nabih N Shalaby NM El-Karef AA Abou-El-Wafa HS. Insulin growth factor inhibitor as a potential new anti-schistosoma drug: An in vivo experimental study. Biomed Pharmacother. (2017) 95:1346–58. 10.1016/j.biopha.2017.09.015 28946182

[55] Allam G. Immunomodulatory effects of curcumin treatment on murine schistosomiasis mansoni. Immunobiology. (2009) 214:712–27. 10.1016/j.imbio.2008.11.017 19249123

[56] Al-Olayan EM El-Khadragy MF Alajmi RA Othman MS Bauomy AA Ibrahim SR. Ceratonia siliqua pod extract ameliorates *Schistosoma mansoni*-induced liver fibrosis and oxidative stress. BMC Complement Altern Med. (2016) 16:434–44. 10.1186/s12906-016-1389-1 27821159 PMC5100080

