# Supplementary figures and images for: The Effect of Different Formulations of Praziquantel in Reducing Worms in the Prepatent Period of Schistosomiasis in Murine Models
Source: Front Public Health. 2022 May 27;10:848633. doi: 10.3389/fpubh.2022.848633 (PMC9184718; doi:10.3389/fpubh.2022.848633)

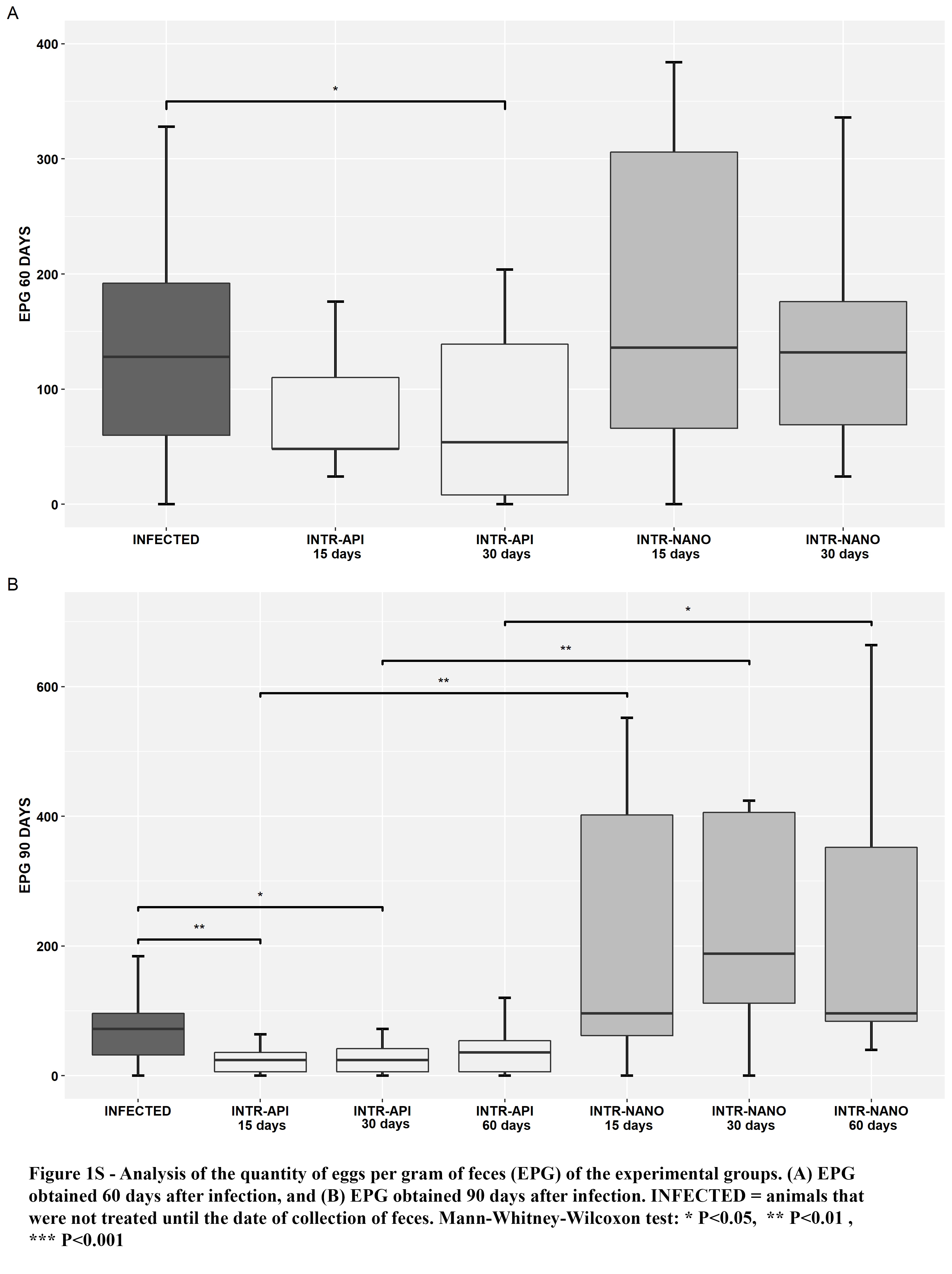

Supplement: Supplementary file 1 [file Image_1.JPEG]

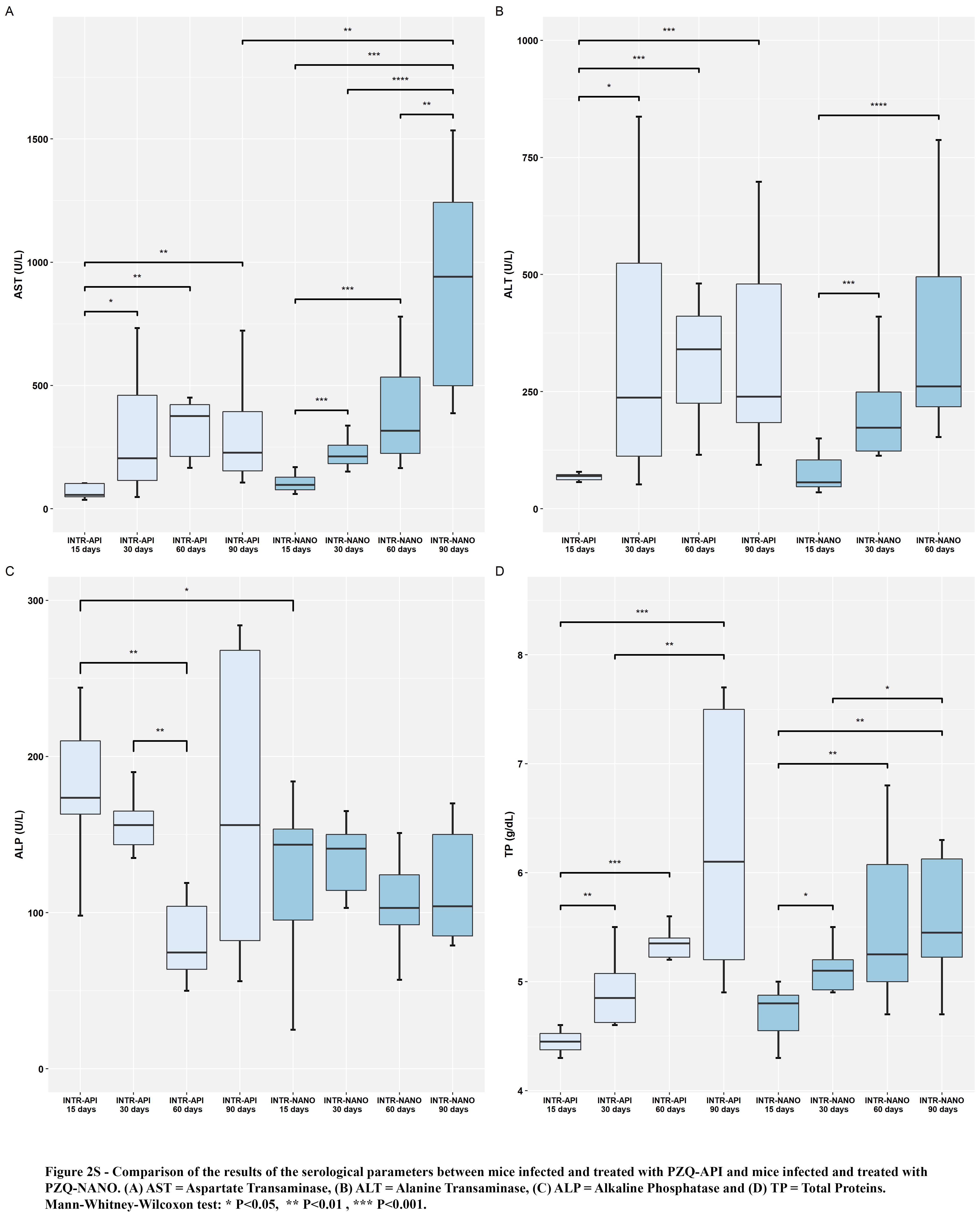

Supplement: Supplementary file 2 [file Image_2.JPEG]
